# Pig liver esterases PLE1 and PLE6: heterologous expression, hydrolysis of common antibiotics and pharmacological consequences

**DOI:** 10.1038/s41598-019-51580-4

**Published:** 2019-10-29

**Authors:** Qiongqiong Zhou, Qiling Xiao, Yongliang Zhang, Xiliang Wang, Yuncai Xiao, Deshi Shi

**Affiliations:** 1 State Key Laboratory of Agricultural Microbiology, College of Veterinary Medicine, Huazhong Agricultural University, Wuhan, 430070 Hubei China; 2 Key Laboratory of Development of Veterinary Diagnostic Products of Ministry of Agricultural, College of Veterinary Medicine, Huazhong Agricultural University, Wuhan, 430070 Hubei China; 3 Key Laboratory of Preventive Veterinary Medicine in Hubei Province, College of Veterinary Medicine, Huazhong Agricultural University, Wuhan, 430070 Hubei China

**Keywords:** Hydrolases, Molecular medicine

## Abstract

Carboxylesterases, historically referred as non-specific esterases, are ubiquitous hydrolases with high catalytic efficiency. Without exceptions, all mammalian species studied contain multiple forms of carboxylesterases. While having been widely studied in humans and experimental animals, these enzymes remain to be characterized in farm animals. In this study, we showed that pig liver esterase 1 (PLE1) and pig liver esterase 6 (PLE6) were highly active toward amoxicillin (AMO) and ampicillin (AMP), two major antibiotics that are widely used in food-supplements. Mass-spectrometric analysis established that the hydrolysis occurred at the β-lactam amide bond and the hydrolysis drastically decreased or completely eliminated the antibacterial activity. Furthermore, hydrolytic activity and proteomic analysis suggested that trace PLEs existed in pig plasma and contributed little to the hydrolysis of AMO and AMP. These results suggested that carboxylesterases-based hydrolysis determines the therapeutic intensity of these and related antibiotics and the magnitude of the determination occurs in a species-dependent manner.

## Introduction

The widespread use of antibiotic supplements in farm animals is a major contributing factor to drug residues, development of drug resistance, and even environmental pollution. While there are many types of antibiotics, β-lactam antibiotics constitute the major type and are effective against both gram-positive and negative bacteria. From 2000 to 2014, the global use of this type of antibioitcs such as AMO and AMP has risen by as much as 35%^1–6^. In humans, 60% of oral doses are excreted unchanged in the urine. Nevetheless, seven metabolites are identified when AMO is incubated with human liver microsomes. These metabolites are formed through oxidation, deamination and conjugation^7–9^.

Carboxylesterases (CEs, E.C.3.1.1.1) constitute a major class of hydrolases with high catalytic efficiency. These enzymes are known to hydrolyze such chemical bonds as carboxylic esters and amides. Therefore, carboxylesterases play critical roles in drug metabolism, and pesticide detoxification^10–17^. Pigs express multiple forms of carboxylesterases with the highest being in the liver. These enzymes are therefore designated as pig liver esterases (PLEs). It has been reported that carboxylesterases demonstrate high enantioselectivity and stereoselectivity. As a result, PLEs are widely used in the area of biosynthesis^18–26^.

AMO and AMP are widely used as growth promoters in animal husbandry including pig farms, and importantly these antibiotics contain an amide bond. We, therefore, tested whether PLEs could hydrolyze these antibiotics. In previous studies, our research group found that PLE1 and PLE6 were the most abundant isoenzymes in Large white and Tongcheng pigs, respectively^27^. Accordingly, in this study, we cloned and expressed two PLEs: PLE1, PLE6, and the recombinant carboxylesterases were tested for the hydrolysis of AMO and AMP. Both PLE1 and PLE6 efficiently hydrolyzed AMO and AMP, and decreased their antibacterial activities. This is the first study to quantify the hydrolytic activities of pure PLEs towards commonly used veterinary drugs.

## Results

### The prepared antibody against PLEs has qualified cross-reactivity

**Figure 1:**
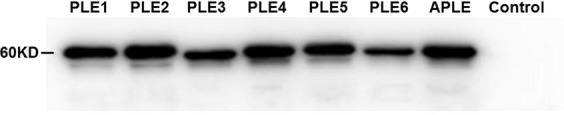
Detection of antibody cross-reactivity against PLEs. Seven recombinant PLEs were detected by western blotting. Control was a sample containing empty vector pET15b.

To determine the cross-reactivity, seven recombinant PLEs were subjected to western blotting analysis. As shown in Fig. 1, this antibody detected a band in all recombinant PLE samples and no band in control (sample transfected empty vector pET15b). The size of the band varied slightly at 60 kDa which was the expected molecular weight of PLE isoenzymes, as the cDNA sequences of PLE isoenzymes contained 1698 base pairs, encoding 566 amino acids with 18AA-signal peptide sequence^23^. These results demonstrated the purified antibody prepared by our research group has qualified cross-reactivity with the seven PLE isoenzymes tested in this study.

### Enzymatic assays of recombinant PLEs and S9 fractions for *para*-nitrophenylacetate (*p*-NPA)

**Figure 2:**
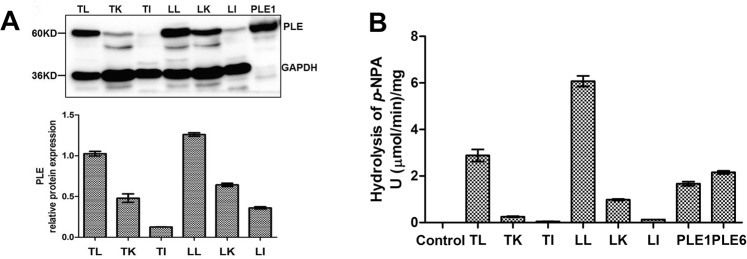
Detection of protein and hydrolytic activities of PLEs and S9 fractions pool. (**A**) Western blotting of PLE in S9 fractions pool (100 μg) from liver, kidney and intestine (n = 3), positive control was recombinant PLE1 (10 μg); (**B**) Hydrolytic activity. Substrate *p*-NPA (200 μM) were prepared in 990 μl of reaction buffer Tris-HCl (50 mM, pH 7.4) and then mixed with protein (10 μg recombinant PLE1 and PLE6 or 100 μg S9 fractions pool). All assays were performed in triplicate with three transfection experiments and the hydrolytic rates were expressed as the mean ± S.D. (T-Tongcheng, L-Large white/Liver, K-Kidney, I-Intestine).

Western blotting detected PLE expression in the liver, kidney and intestine with the liver expressing the highest level, and the molecular weights were consistent with recombinant PLE1 (Fig. 2A). To specify the hydrolytic activity, recombinant PLEs or S9 fractions were incubated with *p*-NPA (a universal substrate for carboxylesterases. The structure of *p*-NPA was shown in Supplementary Fig. S3) and the hydrolysis was spectrophotometarically monitored at 400 nm. Recombinant PLE1 and PLE6 showed the comparable hydrolytic activities of 1.7 U/mg and 2.2 U/mg, respectively (Fig. 2B). Consistent with the high level expression in the liver, S9 fraction from the liver exhibited the highest activity toward *p*-NPA. The liver S9 from Large white pigs had a specific activity of 6.1 U/mg, and the S9 from Tongcheng pigs 2.9 U/mg (Fig. 2B).

### Hydrolysis of AMO and AMP by recombinant PLE1, PLE6 and S9 fractions

**Figure 3:**
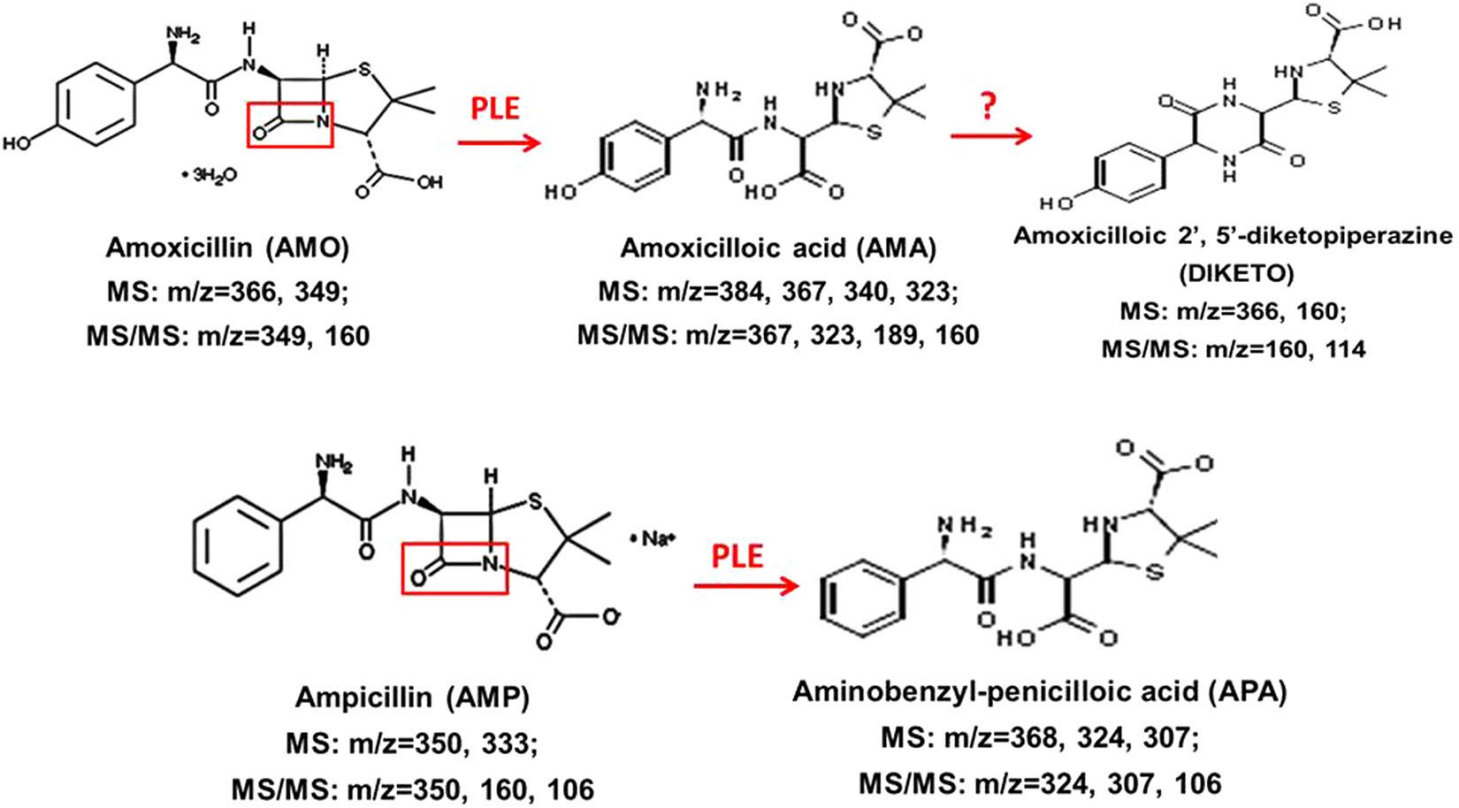
The detail information of parent drugs and metabolites. The parent drugs AMO or AMP and their metabolites AMA, DIKETO and APA were dissolved in DMSO. Acetonitrile was used for dilution of LC-MS/MS simples.

To gain clinical significance, we tested the hydrolysis of AMO and AMP by recombinant PLEs and pig liver S9. The hydrolysis was monitored by LC-MS/MS. As shown in Fig. 3 and Supplementary Table S1, AMO yielded a parent ion at 366 *m/z* and AMP at 350 *m/z*. Incubations of AMO and AMP with PLE1 or PLE6 rapidly decreased the levels of parent drugs. Based on MS and MS/MS spectrograms of product ions (see Supplementary Table S2), AMO produced two major metabolites: AMA (amoxicilloic acid) yielded a parent ion at 384 *m/z* and DIKETO (amoxicilloic 2′,5′-diketopiperazine) at 366 *m/z*, whereas AMP produced a single metabolite: APA (aminobenzyl-penicilloic acid) at 368 *m/z*. The LC-MS/MS method was optimized for the detection of these metabolites with respective standards (the concentration of standards was 4 µg/mL), and the hydrolytic activities of PLEs were quantified with the amount of product. The assay was linear from 0.25 µg/mL to 16 µg/mL for DIKETO, 2 to 64 µg/mL for AMA, 0.5 µg/mL to 32 µg/mL for APA, and the regression coefficients for DIKETO, AMA, and APA were 0.990, 0.999, and 0.999 respectively.

**Figure 4:**
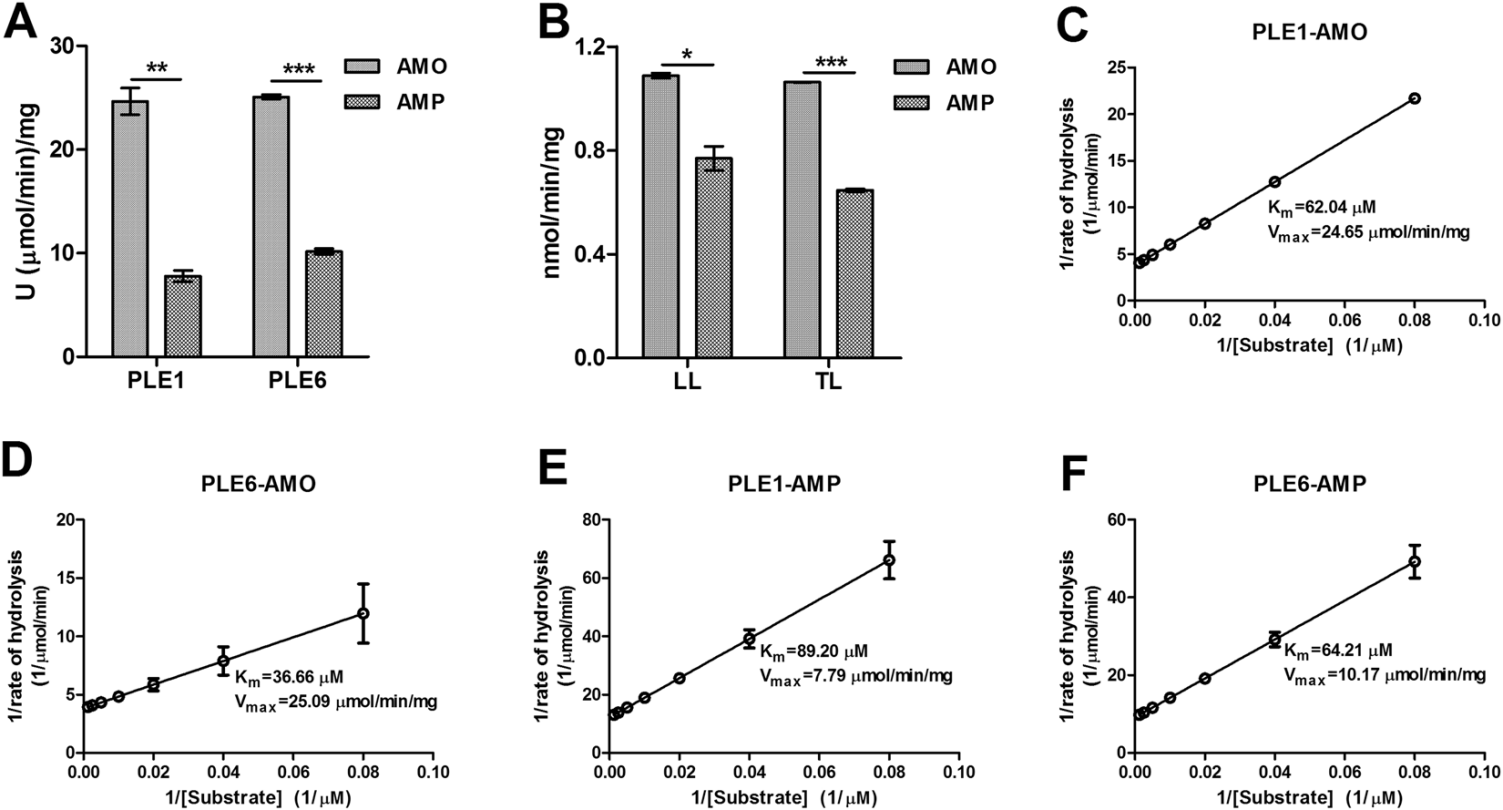
Hydrolysis of AMO/AMP by PLE1, PLE6 and liver S9 fractions and determination of enzyme kinetics. (**A**,**B**) LC-MS/MS detection of the hydrolytic activities of recombinant PLEs and liver S9 fractions toward AMO/AMP. (**C–F**) Line weaver-Burk plot of AMO/AMP hydrolysis by recombinant PLEs. *K*_*m*_ and *V*_*max*_ were calculated by Visual Enzymics. All assays were performed in triplicate with three experiments and the hydrolytic activities are expressed as the mean ± S.D. The asterisk sign denotes statistical significance (^*^P < 0.05; ^**^P < 0.01; ^***^P < 0.001).

Both AMO and AMP were rapidly hydrolyzed by PLE1 and PLE6 to produce AMA and APA respectively. The time-course incubations showed that the amount of parent drug decreased accompanied by increasing the formation of the corresponding metabolites. In addition, liver S9 fractions from both Large white and Tongcheng pigs also hydrolyzed AMO and AMP and produced two major metabolites: DIKETO and APA. DIKETO is an isomer of AMO and more stable than AMA. Likewise, the hydrolysis by liver S9 fractions occurred in a time-dependent manner. PLE1 was more active toward AMO (25.94 U/mg) than AMP (8.34 U/mg), and PLE6 was also more active toward AMO (25.32 U/mg) than AMP (10.44 U/mg) (Fig. 4A). The hydrolytic activities of the Large white pig liver S9 fractions for AMO and AMP were 1.1 nmol/mg/min and 0.8 nmol/mg/min, respectively. Tongcheng pig liver S9 fractions showed comparable activities toward these two antibiotics with a specific activity of 1.1 nmol/mg/min and 0.7 nmol/mg/min, respectively (Fig. 4B).

To gain kinetic insight, the kinetic parameters were determined with PLE1 and PLE6. The hydrolytic rate of AMO/AMP was determined as a function of substrate concentrations (12.5–800 µM). As shown in Fig. 4C–F, data from PLE1 and PLE6 yielded a linear Line weaver-Burk plot. Both PLE1 and PLE6 showed a comparable *V*_*max*_ value toward AMO: 24.65 µmol/mg/min and 25.09 µmol/mg/min, respectively. But PLE6 exhibited a higher *V*_*max*_ values toward AMP (10.17 µmol/mg/min) than PLE1 (7.79 µmol/mg/min). PLE1 had a *K*_*m*_ value of 62.04 µM toward AMO and 89.20 µM toward AMP. In contrast, PLE6 had a lower *K*_*m*_ value than PLE1 for both antibiotics with 36.66 µM toward AMO and 64.21 µM toward AMP. These results suggested that PLE6 was kinetically favorable than PLE1 for both AMO and AMP. Between two antibiotics, AMO was kinetically favorable. According to these results, we concluded that the overall hydrolysis depends on a carboxylesterase as well as a substrate.

### Antibacterial activity

**Figure 5:**
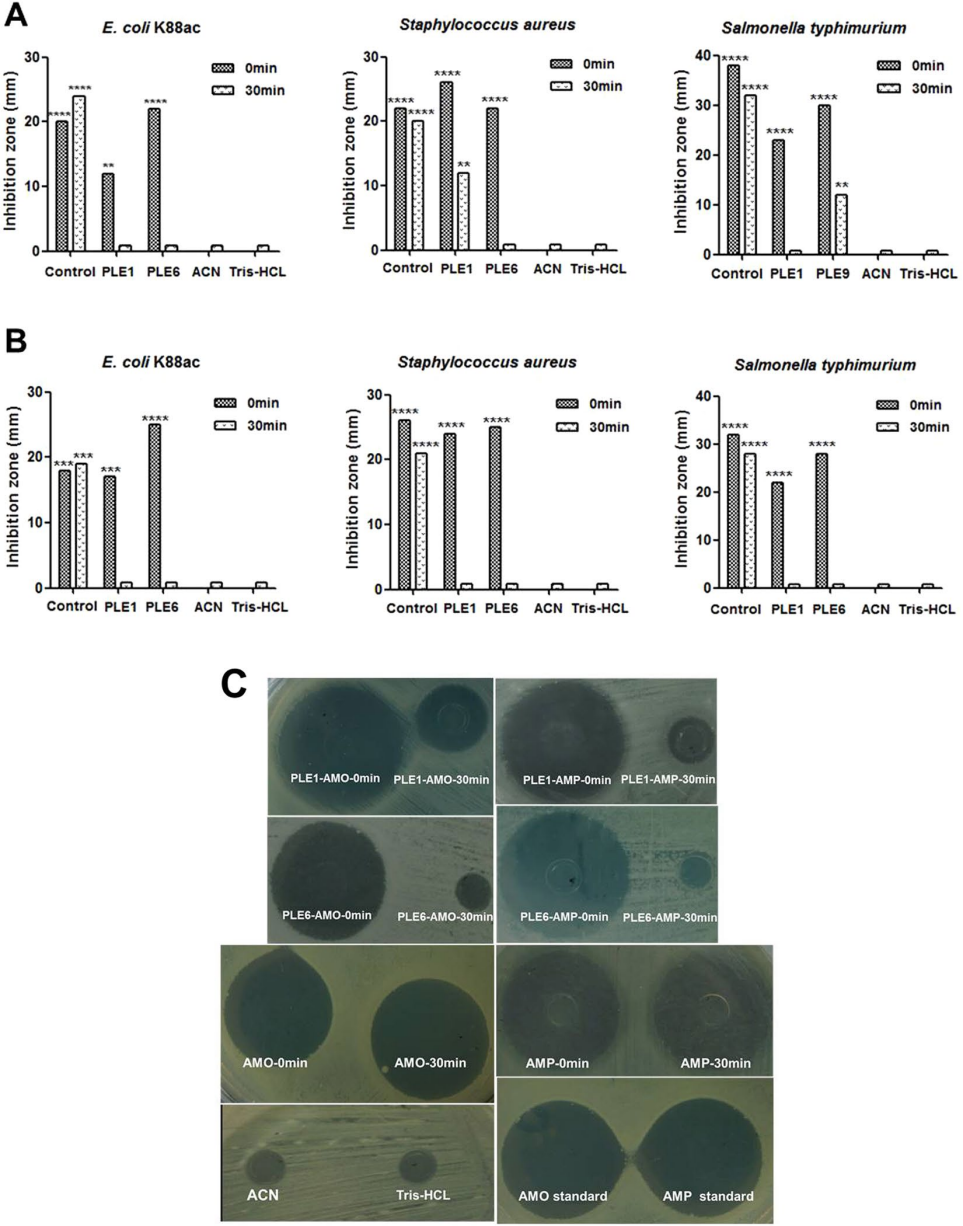
Antibacterial activities of AMO, AMP or metabolites hydrolyzed by PLEs. (**A**) The antibacterial activity of AMO or products AMA hydrolyzed by PLE1 or PLE6. (**B**) The antibacterial activity of AMP or products APA hydrolyzed by PLE1 or PLE6. (**C**) The partially inhibition zone diagrams of AMO, AMP or metabolites hydrolyzed by PLEs for *Salmonella typhimurium*. The antibacterial activities of parent or daughter drugs were showed by diameter of inhibition zones. The diameter of inhibition zone >20 mm is extremely sensitive showed by ****, 15–20 mm is highly sensitive showed by ***, 10–15 mm is moderately sensitive showed by **, <10 mm is slightly sensitive, ≤0 mm is insensitive.

Next we tested the impact of hydrolysis on the antibacterial activity. AMO and AMP showed strong antibacterial activity toward *E. coli K88ac*, *Staphylococcus aureus*, and *Salmonella typhimurium* (Fig. 5A,B). However, incubations with recombinant PLE1 or PLE6 sharply decreased or even eliminated their antibacterial activities. Figure 5C shows the images of the inhibition zones. These results suggested that the therapeutic efficacy of these two antibiotics *in vivo* may be subject to the activity of PLEs.

### PLEs contribute to the intracellular hydrolysis of AMO and AMP

**Figure 6:**
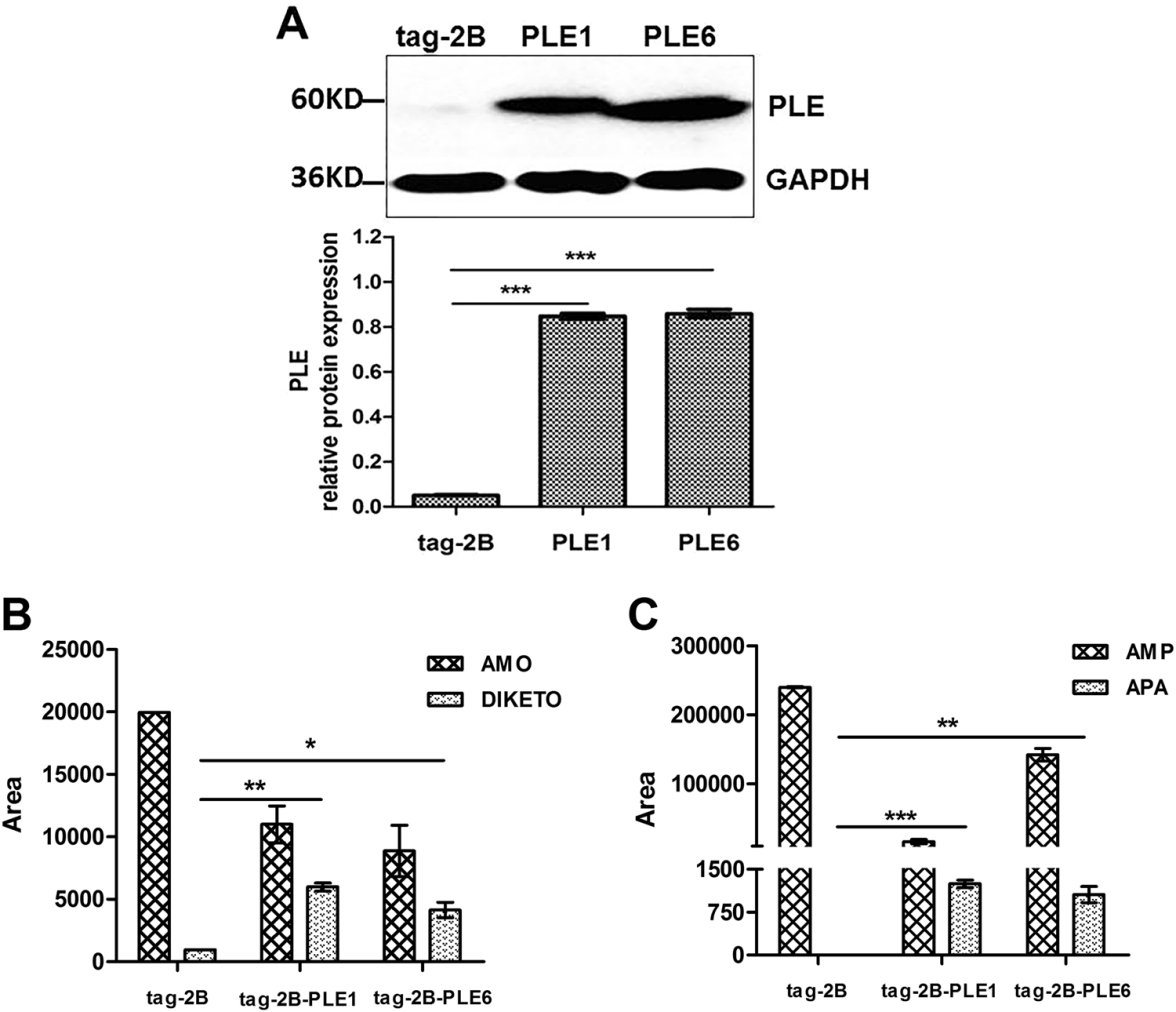
The hydrolytic activities of PLE1 and PLE6 expressed in 293T cells toward AMO and AMP. A: 293T cells were transfected with empty vector tag-2B, tag-2B-PLE1, tag-2B-PLE6, and the cell lysates were analyzed by western blotting. B: The transfected 293T cells were treated with AMO or AMP and the culture supernatants were analyzed by LC-MS/MS. All assays were performed in triplicate with three transfection experiments and the hydrolytic activities are expressed as the mean ± S.D. The asterisk sign denotes statistical significance (^*^P < 0.05; ^**^P < 0.01; ^***^P < 0.001).

To gain the intracellular significance of the hydrolysis, 293T cells were transfected with PLE1, PLE6 or the empty vector (tag-2B) and the hydrolytic activity toward AMO and AMP was determined. As expected, transfection with a PLE construct yielded robust expression (Fig. 6A). In addition, the LC-MS/MS detected the metabolites in the supernatants when the cells were treated with AMO or AMP. The hydrolytic activities of PLE1 toward AMO and AMP were mildly higher than PLE6 (Fig. 6B,C). These results established that PLE1 and PLE6 contributed to the intracellular hydrolysis of AMO and AMP. It was concluded that PLEs expressed in eukaryote cells had a similar hydrolytic activity with PLEs of prokaryotic expression.

### Hydrolysis of AMO and AMP by pig hepatocytes

**Figure 7:**
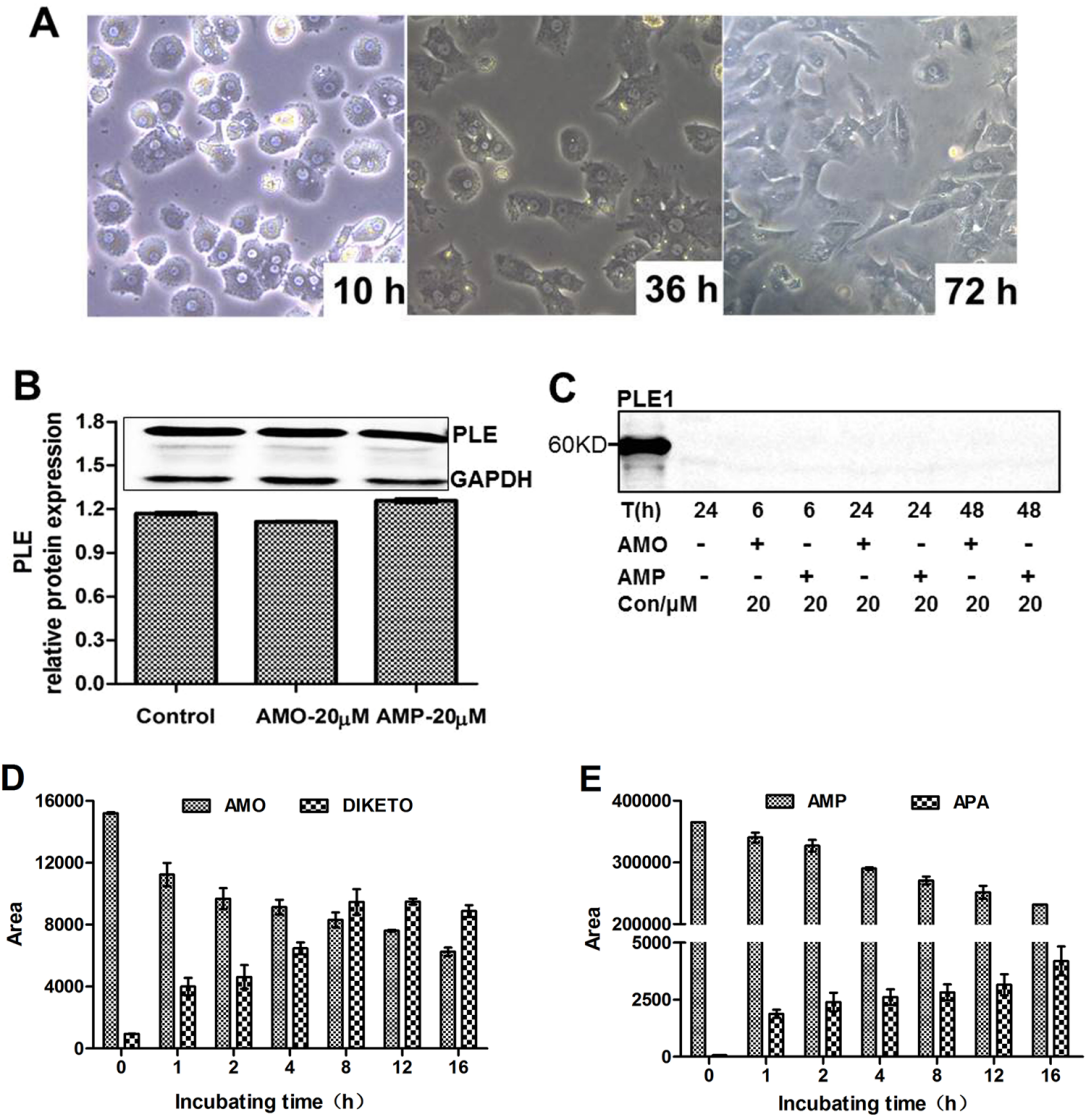
Detection of PLE isoenzymes and the hydrolytic activities in pig hepatocytes toward AMO and AMP. (**A**) Morphological analysis of pig hepatocytes cultured for 10 h, 36 h, and 72 h. (**B**) Western blotting analysis of pig hepatocytes lysate treated with AMO or AMP (20 µM) for 24 h. Control was pig hepatocytes without any treatment. (**C**) Western blotting analysis of supernatants of pig hepatocytes treated with AMO or AMP (20 µM). (**D,E**) LC-MS/MS detection of supernatants of pig hepatocytes treated with AMO or AMP (20 µM). All assays were performed in triplicate with three transfection experiments and the hydrolytic activities are expressed as the mean ± S.D.

To mimic the hydrolysis *in vivo*, pig hepatocytes were prepared and tested for the hydrolysis of AMO and AMP. Hepatocytes from 25-day old male Large white pigs were cultured for 10 h, 36 h, or 72 h, as shown in Fig. 7A. These hepatocytes were healthy and of high purity. These hepatocytes expressed high-levels of PLEs and the expression was not altered by AMO or AMP (Fig. 7B). In addition, no PLE was detected in the supernatants of hepatocytes treated with AMO or AMP for 6 h, 24 h, or 48 h (Fig. 7C), which suggested that PLEs location was not affected by the treatment of AMO or AMP.

To link the expression to catalytic activity, hepatocytes were treated with AMO or AMP and the levels of the metabolites in the supernatants were monitored. As shown in Fig. 7D,E, the levels of the parent drugs decreased whereas the formation of the metabolites increased in a time-dependent manner. It was interesting to note that there was no AMA but DIKETO in the supernatants when the hepatocytes or 293T cells transfected with PLEs were treated with AMO. These results suggested that: maybe because of the instability, AMA was transformed to DIKETO by other enzymes after hydrolysis by PLEs. Taken together, we concluded that AMO and AMP could enter into cells and were effectively hydrolyzed by PLEs.

### Trace PLEs exist in pig serum

**Figure 8:**
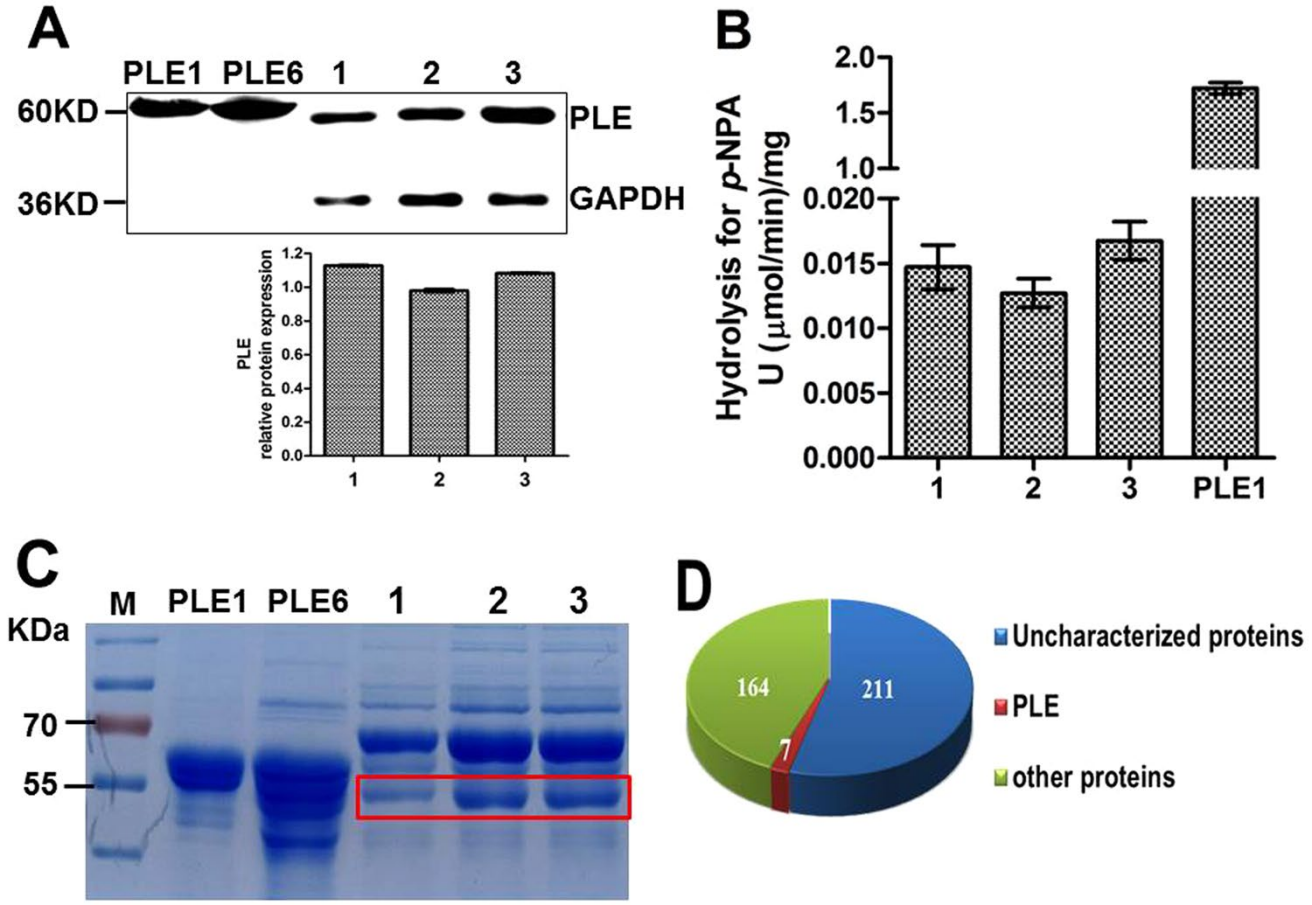
Detection the protein level of PLEs in pig serum and hydrolytic activity for *p*-NPA. (**A**) Western blotting of PLEs in serum pool samples. (**B**) Hydrolytic activity of pig serum for *p*-NPA. (**C**) SDS-PAGE detection of pig serum pool samples. Lane 1, 2 and 3 were serum pool samples (n = 4) from pigs in three different farms respectively and were diluted 100-fold with PBS (50 mM, pH 7.4), the recombinant PLE1 and PLE6 were used as positive controls. The gel piece in the red box was collected for shotgun proteomic analysis. (**D**) 382 kinds of proteins were detected by shotgun proteomic analysis, seven proteins belonged to the PLE family, 164 were known proteins, and 211 were unknown proteins in the UniProt database.

In addition, the question as to whether PLE isoenzymes are presented in pig serum was also explored. Western blotting analysis showed that all serum samples contained a band located at the same position, which was smaller in size than that of recombinant PLE1 and PLE6 (Fig. 8A). Figure 8B showed that serum samples from three different farms had very little hydrolytic activities for *p*-NPA. Furthermore, a shotgun proteomics analysis also detected a very low level of PLE isoenzymes in pig serum (Fig. 8C,D, Table 1). Importantly, LC-MS/MS analysis showed the serum samples could not hydrolyze AMO or AMP (data not shown). This discrepancy was likely due to the fact that there are some other proteins in serum which share with PLEs in certain part of the amino sequences. On the basis of these results, it was concluded that only trace PLEs existed in pig serum and the trace PLE in serum did not contribute significantly to the hydrolysis of either antibiotic.

**Table 1:** The information of PLE proteins in pig serum by shotgun proteomic analysis.

Accession number/Gene name	pI/Mw (kDa)	No. of (unique) peptides	Cover percent (%)	Protein description	Unique peptide sequences
A0A1S6L960/PLE1	5.62/62077.69	1/1	2.12	pig Carboxylic ester hydrolase	GDAPEEEVSLSK
A0A1S6L967/PLE-B9	5.70/62008.42	1/1	2.12		
F1RF15/CES1	5.53/53181.31	1/1	2.49		
A0A1S6L948/PLE-C4	5.78/61976.39	1/1	2.12		
A0A1S6L959/PLE-G2	5.61/61965.38	1/1	2.12		
Q29550/PLE5	5.62/62015.53	1/1	2.12	liver carboxylesterase	
A9GYW6/APLE	5.78/59961.2	1/1	2.19	pig carboxylic ester hydrolase	

## Discussion

The important roles that carboxylesterases play in the pharmacokinetics of many therapeutic agents have been well characterized in humans^10–12,14–17,28–33^. Pig liver esterases (PLEs) belong to mammal carboxylesterase, despite extensive use in organic chemistry synthesis due to their high stereoselectivity and hydrolytic activity^22,26,34,35^, little is known about their pharmacological roles, particularly in veterinary medicine. In this study, we found that two PLE isoenzymes, PLE1 and PLE6 could hydrolyze AMO and AMP, two antibiotics that are widely used in animal husbandry. Moreover, the overall hydrolytic activity varied depending on enzyme as well as substrate. Our data showed that AMO was more greatly affected by either of the PLEs tested and that AMP was more susceptible to PLE6 than to PLE1. Our published paper already proved that PLE1 and PLE6 were the most abundant isoenzymes in Large white pigs and Tongcheng pigs (outstanding Chinese local breed) respectively^27^. Comprehensively, the expression level and hydrolytic activity difference of PLE isoenzymes most probably were the foundation of clinical rationale administration of antibiotics.

It is well-established that bacteria produce β-lactamase to abolish β-lactam activity. *Jones et al*. also found the β-lactamase activity of pig liver esterase, which was used to synthesize the β-lactams^36^. In this study, the β-lactamase activity of PLE were demonstrated by incubation AMO and AMP with pure PLE isoenzymes, livers S9, pig hepatocytes and PLE transfected 293T cells, and the significance of β-lactamase activity of PLE for antibacterial activities of AMO and AMP were further focused on. β-lactamase activity of PLE were proved to abolish β-lactam activity obviously or even totally, which suggested that the expression level and hydrolytic activity of PLEs were highly correlated with the therapeutic efficacy and metabolism of antibiotic *in vivo*.

There are reports that under aqueous conditions, the hydrolytic products of AMO are pH-sensitive, whereas the hydrolytic products of AMP are pH-insensitive. In addition, the hydrolysis of AMO can be significantly influenced by the presence of Fe^2+^, Zn^2+^, Ca^2+^ and Mg^2+^ in solution^8,37,38^. So, the differences in the bivalent metal ion concentration and pH between the Tris-HCl buffer and cells may be the reason of product differences. But this issue remains to be determined as the dependence on bivalent metal ion occurs with carboxylesterase-based hydrolysis or an involvement of other hydrolases. In this study, LC-MS/MS analysis demonstrated that recombinant PLEs could hydrolyze AMO to produce AMA, while the liver S9, pig hepatocytes and 293T cells transfected with PLEs produce DIKETO. That could be because that after hydrolysis of AMO by PLEs, AMA is unstable in the cells and is transformed to DIKETO by other enzymes. Moreover, LC-MS/MS showed that the levels of AMA increased and then decreased with the increasing of hydrolysis time. Maybe it is because that AMA is converted to DIKETO or other metabolites during hydrolysis, which needs further explore. Consistent with our results, Nägele and Moritz reported that DIKETO was a possible further metabolite of AMO^9^.

Our results suggest that PLEs in various pig tissues play a key role in the metabolism process of AMO and AMP. Consistent with our results, it was reported that the metabolites of AMO and AMP in the liver, kidney, fat and muscle of pig were AMA/DIKETO or APA respectively^39^. However, human liver microsomes metabolized AMO to form six phase I metabolites and one phase II metabolite via oxidation, hydroxylation and oxidative deamination, as well as the combination of these reactions, and the reactions were mainly attributed to the function of cytochrome P450 monooxygenase^40^. Therefore, our data which showed robust hydrolysis of AMO and AMP by PLEs in pig hepatocytes, indicated profound species differences in carboxylesterases-based drug metabolism.

In addition, we explored the possibility that circulating levels of PLEs might be a factor in antibiotic metabolism. Carboxylesterases have been detected in the plasma of cat, mouse, rabbit, rat, and horse, but not in that of humans^41^. In our study, only trace PLEs was detected in pig plasma, which consistent with previous observations in humans. And the trace PLEs in pig serum also had no hydrolytic activity against either AMO or AMP. These results indicate that the hydrolytic activity of PLE *in vivo* is likely to be intracellular and follow antibiotic uptake into cells.

β-lactam antibiotics are the most common oral treatments for both human and pig respiratory disease. AMO is highly effective against *Streptococcus pneumoniae* and *Haemophilus influenza* in humans and against *Haemophilus parasuis* and *Actinobacillus pleuropneumoniae* in pigs^42,43^. It has been reported that the bioavailability of AMO is 70–92% in humans^44^, 31–47% in pigs^45^, and only 5–10% in horses^46^ where plasma carboxylesterase activity is high^41,46^. These findings, in conjunction with our observations, indicate that carboxylesterases-based hydrolysis occurs in a species-dependent manner and that these species differences may have strong clinical significance. In particular, the application of AMO/AMP and other veterinary drugs in pigs or other animals should take account of the impact of hepatic first pass metabolism and PLEs expression profiles, which is likely to reduce the bioavailability of these antibiotics^47^.

## Materials and Methods

### Antibody cross-reactivity

Immunogen of PLE was prepared by conjugating the conserved sequences of PLE isoenzymes (SKEAAKKPPKIKC and CNTQAAKRLKGEE) to keyhole limpet hemocyanin, the rabbit derived PLE polyclonal antibody were prepared and purified as described previously^48^. The cross-activity of the antibody was determined with 7 recombinant PLEs (PLE1 to PLE6, and APLE). The recombinant PLEs were expressed and purified as described by Böttcher *et al*.^18^. In brief, the recombinant plasmid pET-15b-PLE constructed by our research group and molecular chaperone pGro7 were transfected into *E. coli Origami* (DE3), positive clones were selected to culture at 30 °C, 200 rpm. L-arabinose (Sigma) was firstly added to final concentration of 1 mg/mL to induce the expression of pGro7, when the optical density at 600 nm reached 0.6–0.8, IPTG (isopropyl β-D-1-thiogalactopyranoside) (Sigma) was added to final concentration of 40 µM to induce the expression of PLEs. After being cultured for 6 h at 30 °C, 200 rpm, the cells were collected by centrifugation (4 °C, 8000 rpm, 10 min) and broken. The samples were centrifuged for 30 min at 4 °C, 12000 rpm, and the supernatants were harvested and purified by AKTA purifier and His Trap FF crude column. The purified PLEs were analyzed by western blotting.

### Enzymatic assays for *p*-NPA, AMO and AMP

Adult Tongcheng and Large White pigs, no genetic relationship in three generations, were purchased from conservation farms in Tongcheng country and Hubei Tianzhong Animal Husbandry Co., Ltd., Hubei province, China. The liver, kidney and intestine were harvested and frozen in liquid nitrogen immediately. The tissues were used for the preparation of S9 fractions (n = 3, female) as described previously^33^. The study was performed in accordance with the Guide for the Care and Use of Laboratory Animals Monitoring Committee of Hubei Province, China. The protocol was approved by the Committee on the Ethics of Animal Experiments at the College of Veterinary Medicine, Huazhong Agricultural University.

Fresh blood samples were obtained from adult pigs (Duroc × Landrace × Large white, 4 months old approximately, n = 4, male) from three different farms and then were placed at 4 °C for overnight after 1 h at 37 °C. Serum samples from the same farm were mixed in equal amount and diluted with phosphate buffer solution (50 mM, pH 7.4). The protein concentrations were determined by BCA Protein Assay Kit (Pierce) according to the protocol of manufacturer. The hydrolytic activities of pig liver S9, pig serum samples and recombinant PLEs for *p*-NPA was monitored by spectrometer as described previously^16^.

The hydrolysis of AMO and AMP (Sigma, USA) was carried out at 37 °C in a total volume of 100 μL at a final concentration of 12.5, 25, 50, 100, 200, 400, 800 μM, respectively. The final concentration of the organic solvent in all reaction was 0.1% (v/v). AMO or AMP was added to the Tris-HCl buffer (50 mM, pH 7.4), and the hydrolysis reaction was initiated by adding enzyme preparation (10 μg PLE1 or PLE6), S9 fraction (100 μg) or serum samples (200 μg). The reaction lasted for 0.5 min, 15 min, 30 min, 45 min or 90 min respectively and then terminated by adding acetonitrile (200 μL). The reaction mixture was placed in an ice-bath for 10 min and then centrifuged at 12,000 rpm for 15 min at 4 °C. The supernatant (200 μL) was removed and diluted with acetonitrile (800 μL), following which, the parent drug and metabolites were monitored by LC-MS/MS. Control samples were either incubated with thermally inactivated enzymes (treated at 100 °C for 10 min) or the enzymatic reactions were stopped at 0 min.

### LC-MS/MS

The LC-MS/MS analysis was performed using a Waters Xevo G2 QTOF mass spectrometer and a Waters Acquity Ultra Performance Liquid Chromatography (Waters, USA), with a WATERS ACQUITY UPLC/XEVO G2 QTOF system. Various parameters were adjusted to obtain the optimal conditions for the quantification of AMO/AMP and their metabolites. Chromatographic separation was performed using a reverse-phase C18 polymeric column (ACQUITY UPLC BEH, 2.1 mm × 100 mm, 1.7 μm). Gradient elution was performed using a mobile phase of 0.1% formic acid in water (A) and acetonitrile (B) at a flow rate of 0.4 mL/min. The gradient elution used was as follows: 0–0.5 min, A-99%; 0.6–7.5 min, A-1%; 7.6–10 min, A-99%. The auto sampler was set at 6 °C and the column temperature was 45 °C. The scanned area λ was 190–600 nm and covered almost all spectral ranges^8,9,49^. Conditions for the ESI source for positive ionization mode were optimized by direct infusion of standard solutions of AMO, AMP, AMA, DIKETO and APA (Toronto Research Chemicals). Capillary voltage was set to 2 kv, sampling cone was 30 v and source temperature was 120 °C. N_2_ was used as the desolvation gas with 800 L/h at 450 °C, and Argon was used as the collision gas with 50 L/h. The collision energy was set to 6.0 ev and the scanning range was *m/z* = 100–1000. Data analysis was performed with MassLynx software Version 4.1.

All quantifications were performed based on the peak areas. The calibration curves were constructed with concentration (x axis) plotted against peak area (y axis). All solutions and reagents used for LC-MS/MS were of mass spectrum purity and purchased from Thermo-Fisher Scientific.

### Oxford cup bacteriostatic test

To specify the effect of hydrolysis on the inhibitory activity (antibiotic), the oxford cup antibacterial test was performed. The indicator bacterial strains were *Staphylococcus aureus* (ATCC25923), *Salmonella typhimurium* (CVCC542), and *E. coli K88ac* (C83905). Bacteria were cultured to the logarithmic phase, adjusted to 1–10 × 10^7^ *CFU/mL*, and spread uniformly on an LB agar plate. Thereafter hydrolytic reaction mixture (200 μL) of AMO or AMP incubated with recombinant PLEs for 0 min or 30 min or control (no PLEs) was added to oxford cup. The incubations were prepared at a drug solution final concentration of 10 μg/200 μL as described above. The incubation mixture was centrifuged at 12,000 rpm for 15 min at 4 °C prior to applying to the plates. The plates were placed at 4 °C for 4 h, and then transferred to a 37 °C incubator for 12–16 h. The diameter of the bacteriostatic circles was recorded with Vernier caliper. Acetonitrile (solvent), Tris-HCL, and AMO/AMP standard solutions were used as the negative or positive controls.

### Transient transfection

The cDNA sequences encoding PLE1 and PLE6 were sub-cloned into the pCMV-tag-2B vector with primers shown in Table 2. 293T cells were cultured and transfected with Lipofectamine 2000 Transfection Reagent (Invitrogen) according to Shi *et al*.^16^. The expression was monitored by western blotting.

**Table 2:** The primers for sub-cloning cDNA of PLE1 and PLE6.

Primers	Sequences 5′-3′
pCMV-tag-2B-PLE (up)	GAATTCATGTGGCTTCTCCCGCTGGTCCTGACCTCCCTCG
pCMV-tag-2B-PLE (down)	CTCGAGTCACAGCTCAGCATGCTTTATCTTGGGTGGCTTCTTTGCT

### Treatment of pig hepatocytes with AMO or AMP

Pig hepatocytes were isolated from the liver of 25-day old healthy male Large white pigs. The isolation and culture were performed according to Zhou *et al*.^50^ with slight modification. The pig hepatocytes were cultured in Dulbecco’s modified Eagle’s medium with 10% fetal calf serum. Thereafter cells were treated with AMO or AMP at a final concentration of 20 µM and cultured at 37 °C in a humid atmosphere of 5% CO_2_ for 1, 2, 4, 8, 12, 16, or 24 h respectively. The supernatants were collected and mixed well with equal volume of acetonitrile, which were analyzed by LC-MS/MS. In addition, the supernatants and the cell lysates were analyzed by western blotting.

### Shotgun proteomic analysis

Serum samples from the same farm were mixed in equal amount, and then mixed well with 5 × loading buffer and boiled for 10 min. The samples were electrophoretically separated in polyacrylamide gels (12%). Protein bands were visualized by Coomassie Blue R-250 staining, and were cut and analyzed by shotgun proteomic.

The gel pieces contained the protein bands were rinsed thrice using Milli-Q water and destained with 100 mM NH_4_HCO_3_ (Sigma) in 30% acetonitrile at 37 °C until the color depigmented completely. After dried in a vacuum centrifuge, the in-gel proteins were reduced with 100 mM NH_4_HCO_3_ in 10 mM dithiothreitol (Sigma) for 90 min at 37 °C. Then, gel pieces were alkylated with 60 mM iodoacetamide (Sigma) in 100 mM NH_4_HCO_3_ in the dark at room temperature for 20 minutes after rinsed by 100% acetonitrile. Gel pieces were rinsed with 100 mM NH_4_HCO_3_ for 15 min and 100% acetonitrile for 5 min, respectively, and dried in a vacuum centrifuge. Subsequently, gel pieces were digested in 10 ng/μL chymotrypsin (Promega) for 20 h at 37 °C. The peptides were extracted three times with 0.1% trifluoroacetic acid (Sigma) in 60% acetonitrile. The extracts were evaporated in a vacuum centrifuge, and resuspended with 0.1% formic acid (Sigma) to detect by HPLC separation and MS analysis.

After chymotrypsin digested, all digested peptide mixtures were separated by HPLC and analyzed by tandem MS. Samples were loaded onto a C18 reverse phase trap column (Thermo Scientific Acclaim PepMap100, 2 cm × 100 μm) and analyzed with a C18-reversed phase analytical column (Thermo Scientific Easy Column, 10 cm × 75 μm, 3 μm). Gradient elution was performed using buffer A (0.1% formic acid) and buffer B (84% acetonitrile, 0.1% formic acid) at a flow rate of 0.3 μL/min. The gradient elution used was as follows: 0–35% buffer B for 50 min; 35–100% buffer B for 5 min; hold in 100% buffer B for 5 min. A Q exactive mass spectrometer (Thermo Scientific) was used for MS/MS in positive ion mode. Automatic gain control (AGC) target was set to 1e^6^ and maximum inject time was 50 ms. Dynamic exclusion duration was 60 s. Survey scans were acquired at a resolution of 70,000 at *m/z* = 300–1800, MS_2_ activation type was high energy collision dissociation (HCD) whose resolution was set to 17,500 at *m/z* = 200–2000, and isolation width was 2 *m/z*. Normalized collision energy was 30 ev and the underfill ratio was defined as 0.1%.

### Western blotting analysis

Protein expression levels were determined using western blotting. Equal amount of proteins were separated on polyacrylamide gels (12.5%) with a stacking gel (5%) in a mini-gel apparatus and transferred electrophoretically to a PVDF membrane. The blots were incubated with the purified rabbit antiserum against PLE or GAPDH (glyceraldehyde-3-phosphate dehydrogenase, the house keeping gene) primary antibody respectively. The primary antibody against PLEs was prepared by our previous study^27^, while the primary antibody against GAPDH was purchased from Proteintech (China). The second antibody was goat anti-rabbit IgG conjugated with horseradish peroxidase. The signal was captured by MF-Chemi BIS Chemiluminescence imager (Bio-Imaging Systems) and the relative intensities were quantified by Image J software.

### Statistical analysis

Data were presented as mean ± standard deviation (SD) of at least three independent experiments. Statistical analysis was carried out using one-way analysis of variance, followed by T test. Statistical significance was made at P < 0.05 and indicated by asterisks (^*^P < 0.05; ^**^P < 0.01; ^***^P < 0.001).

## Supplementary information

Supplementary information

## References

[1] Huerta B, Marti E, Gros M, Armengol J (2013). Exploring the links between antibiotic occurrence, antibiotic resistance, and bacterial communities in water supply reservoirs. Science of the Total Environment.

[2] Kümmerer K (2003). Significance of antibiotics in the environment. J Antimicrob Chemother.

[3] Manzetti S, Ghisi R (2014). The environmental release and fate of antibiotics. Marine Pollution Bulletin.

[4] Van Boeckel TP (2014). Global antibiotic consumption 2000 to 2010: an analysis of national pharmaceutical sales data. Lancet Infectious Diseases.

[5] Gordon C, Regamey C, Kirby WM (1972). Comparative clinical pharmacology of amoxicillin and ampicillin administered orally. Antimicrobial Agents & Chemotherapy.

[6] Xu Y (2016). Occurrence and distribution of antibiotics, antibiotic resistance genes in the urban rivers in Beijing, China. Environmental Pollution.

[7] Arancibia A, Guttmann J, González G, González C (1980). Absorption and disposition kinetics of amoxicillin in normal human subjects. Antimicrobial Agents & Chemotherapy.

[8] Gozlan I, Rotstein A, Avisar D (2013). Amoxicillin-degradation products formed under controlled environmental conditions: Identification and determination in the aquatic environment. Chemosphere.

[9] Nägele E, Moritz R (2005). Structure elucidation of degradation products of the antibiotic amoxicillin with ion trap MS n and accurate mass determination by ESI TOF. Journal of the American Society for Mass Spectrometry.

[10] Dean RA, Christian CD, Sample RH, Bosron WF (1991). Human liver cocaine esterases: Ethanol-mediated formation of ethylcocaine. Faseb Journal Official Publication of the Federation of American Societies for Experimental Biology.

[11] Hosokawa M (2008). Structure and catalytic properties of carboxylesterase isozymes involved in metabolic activation of prodrugs. Molecules.

[12] Redinbo MR, Potter PM (2005). Mammalian carboxylesterases: from drug targets to protein therapeutics. Drug Discovery Today.

[13] Satoh T, Hosokawa M (1998). The mammalian carboxylesterases: from molecules to functions. Annu Rev Pharmacol Toxicol.

[14] Satoh T, Hosokawa M (2006). Structure, function and regulation of carboxylesterases. Chemico-Biological Interactions.

[15] Brzezinski MR, Abraham TL, Stone CL, Dean RA, Bosron WF (1994). Purification and characterization of a human liver cocaine carboxylesterase that catalyzes the production of benzoylecgonine and the formation of cocaethylene from alcohol and cocaine. Biochemical Pharmacology.

[16] Shi D (2006). Anti-influenza prodrug oseltamivir is activated by carboxylesterase human carboxylesterase 1, and the activation is inhibited by antiplatelet agent clopidogrel. Journal of Pharmacology & Experimental Therapeutics.

[17] Xie M, Yang D, Wu M, Xue B, Yan B (2003). Mouse liver and kidney carboxylesterase (M-LK) rapidly hydrolyzes antitumor prodrug irinotecan and the N-terminal three quarter sequence determines substrate selectivity. Drug Metabolism & Disposition.

[18] Böttcher D, Brüsehaber E, Doderer K, Bornscheuer UT (2007). Functional expression of the gamma-isoenzyme of pig liver carboxyl esterase in Escherichia coli. Applied Microbiology & Biotechnology.

[19] David L, Guo XJ, Villard C, Moulin A, Puigserver A (1998). Purification and molecular cloning of porcine intestinal glycerol-ester hydrolase–evidence for its identity with carboxylesterase. European Journal of Biochemistry.

[20] Brüsehaber E, Schwiebs A, Schmidt M, Böttcher D, Bornscheuer UT (2010). Production of pig liver esterase in batch fermentation of *E. coli* Origami. Applied Microbiology & Biotechnology.

[21] Hasenpusch D, Bornscheuer UT, Langel W (2011). Simulation on the structure of pig liver esterase. Journal of Molecular Modeling.

[22] Junge W, Heymann E (1979). Characterization of the Isoenzymes of Pig-Liver Esterase 2. Kinetic Studies. Febs Journal.

[23] Lange S, Musidlowska A, Schmidt-Dannert C, Schmitt J, Bornscheuer UT (2001). Cloning, Functional Expression, and Characterization of Recombinant Pig Liver Esterase. Chembiochem A European Journal of Chemical Biology.

[24] Morris AP, Brain KR, Heard CM (2008). Synthesis of haloperidol prodrugs and their hydrolysis by porcine liver esterase. Drug Metabolism Letters.

[25] Oboh OT, Lamango NS (2008). Liver Prenylated Methylated Protein Methyl Esterase Is the Same Enzyme as Sus scrofa Carboxylesterase. Journal of Biochemical & Molecular Toxicology.

[26] Smith ME (2012). Investigation of the Cosolvent Effect on Six Isoenzymes of PLE in the Enantioselective Hydrolysis of Selected α,α-Disubstituted Malonate Esters. Chemcatchem.

[27] Xiao, Q., *et al* Breed Differences in Pig Liver Esterase (PLE) between Tongcheng (Chinese Local Breed) and Large White Pigs. *Scientific Reports* **8** (2018). 10.1038/s41598-018-34695-y PMC6218520 30397234

[28] Hosokawa M, Hirata K, Nakata F, Suga T, Satoh T (1994). Species differences in the induction of hepatic microsomal carboxylesterases caused by dietary exposure to di(2-ethylhexyl)phthalate, a peroxisome proliferator. Drug Metabolism & Disposition the Biological Fate of Chemicals.

[29] Krishnasamy S, Gross NJ, Teng AL, Schultz RM, Dhand R (1997). Lung “surfactant convertase” is a member of the carboxylesterase family. Biochem Biophys Res Commun.

[30] Lian J, Nelson R, Lehner R (2018). Carboxylesterases in lipid metabolism: from mouse to human. Protein & Cell.

[31] Quiroga AD (2012). Deficiency of carboxylesterase 1/esterase-x results in obesity, hepatic steatosis, and hyperlipidemia. Hepatology.

[32] Shi D, Yang D, Prinssen EP, Davies BE, Yan B (2011). Surge in expression of carboxylesterase 1 during the post-neonatal stage enables a rapid gain of the capacity to activate the anti-influenza prodrug oseltamivir. Journal of Infectious Diseases.

[33] Yang J, Shi D, Yang D, Song X, Yan B (2007). Interleukin-6 alters the cellular responsiveness to clopidogrel, irinotecan, and oseltamivir by suppressing the expression of carboxylesterases HCE1 and HCE2. Molecular Pharmacology.

[34] Brüsehaber E, Böttcher D, Bornscheuer UT (2009). Insights into the physiological role of pig liver esterase: isoenzymes show differences in the demethylation of prenylated proteins. Bioorganic & Medicinal Chemistry.

[35] Roberti M (2000). Pig liver esterase (PLE)-mediated resolution of N-substituted 4-benzoyloxy-3-carbomethoxypiperidines: a convenient preparation of 4-hydroxy- and 4-benzoyloxy-3-carbomethoxypiperidines in enantiomerically pure form. Tetrahedron Asymmetry.

[36] Jones, M. & Page, M. I. An esterase with β-lactamase activity. *Journal of the Chemical Society, Chemical Communications*, 316–317 (1991).

[37] Lamm A, Gozlan I, Rotstein A, Avisar D (2009). Detection of amoxicillin-diketopiperazine-2′,5′ in wastewater samples. Journal of Environmental Science & Health Part A Toxic/hazardous Substances & Environmental Engineering.

[38] Wang J (1986). Epimerization of Amoxicillin Piperazine-2,5-dione in Acidic Solutions. Chemical & Pharmaceutical Bulletin.

[39] Reyns T, Cherlet M, De Baere S, De Backer P, Croubels S (2008). Rapid method for the quantification of amoxicillin and its major metabolites in pig tissues by liquid chromatography-tandem mass spectrometry with emphasis on stability issues. Journal of Chromatography B.

[40] Szultka M, Krzeminski R, Jackowski M, Buszewski B (2014). Identification of *In Vitro* Metabolites of Amoxicillin in Human Liver Microsomes by LC–ESI/MS. Chromatographia.

[41] Li B (2005). Butyrylcholinesterase, paraoxonase, and albumin esterase, but not carboxylesterase, are present in human plasma. Biochemical Pharmacology.

[42] Bergogne-Berezin, E., Morel, C., Benard, Y., Bethelot, G. & Kafe, H. Vol. 14 267–272 (1978). 279981

[43] Hagstad H (1984). Penetration of ampicillin (‘Pondocillin’) and amoxycillin (‘Imacillin’) into the bronchial secretions. Pharmatherapeutica.

[44] Paintaud G (1992). Nonlinearity of amoxicillin absorption kinetics in human. European Journal of Clinical Pharmacology.

[45] Lashev L (1986). Comparative pharmacokinetic investigations on amoxicillin in domestic animals. Veterinarnomediciniski Nauki.

[46] Ensink JM, Klein WR, Mevius DJ, Klarenbeck A, Vulto AG (1992). Bioavailability of oral penicillins in the horse: a comparison of pivampicillin and amoxicillin. Journal of Veterinary Pharmacology and Therapeutics.

[47] Wilson WD, Spensley MS, Baggot JD, Hietala SK (1988). Pharmacokinetics and estimated bioavailability of amoxicillin in mares after intravenous, intramuscular and oral administration. American Journal of Veterinary Research.

[48] Zhu W (2000). Dexamethasone differentially regulates expression of carboxylesterase genes in humans and rats. Drug metabolism and disposition: the biological fate of chemicals.

[49] Reyns T, Boever SD, Baere SD, Backer PD, Croubels S (2008). Tissue Depletion of Amoxicillin and Its Major Metabolites in Pigs: Influence of the Administration Route and the Simultaneous Dosage of Clavulanic Acid. Journal of Agricultural & Food Chemistry.

[50] Zhou, X. *et al*. Cecropin B Represses CYP3A29 Expression through Activation of the TLR2/4-NF-κB/PXR Signaling Pathway. *Scientific Reports* **6** (2016). 10.1038/srep27876 PMC4906279 27296244

