# Supplementary material for: Pig liver esterases PLE1 and PLE6: heterologous expression, hydrolysis of common antibiotics and pharmacological consequences
Source: Sci Rep. 2019 Oct 29;9:15564. doi: 10.1038/s41598-019-51580-4 (PMC6820543; doi:10.1038/s41598-019-51580-4)
Supplement: Supplementary file 1 — Supplementary information [file 41598_2019_51580_MOESM1_ESM.docx]

**Title Page**

**Pig liver esterases PLE1 and PLE6: heterologous expression, hydrolysis of common antibiotics and pharmacological consequences**

Qiongqiong Zhou ^1,2,3^, Qiling Xiao ^1,2,3^, Yongliang Zhang ^1,2,3^, Xiliang Wang ^1,2,3^,

Yuncai Xiao ^1,2,3^, Deshi Shi ^1,2,3*^

^1^ State Key Laboratory of Agricultural Microbiology, College of Veterinary Medicine, Huazhong Agricultural University, Wuhan, 430070, Hubei, China.

^2^ Key Laboratory of Development of Veterinary Diagnostic Products of Ministry of Agricultural, College of Veterinary Medicine, Huazhong Agricultural University, Wuhan, 430070, Hubei, China

^3^ Key Laboratory of Preventive Veterinary Medicine in Hubei Province, College of Veterinary Medicine, Huazhong Agricultural University, Wuhan, 430070, Hubei, China.

***Corresponding author:** Deshi Shi ([rock@mail.hzau.edu.cn](mailto:rock@mail.hzau.edu.cn) )

**Supplementary Figure S1**

**
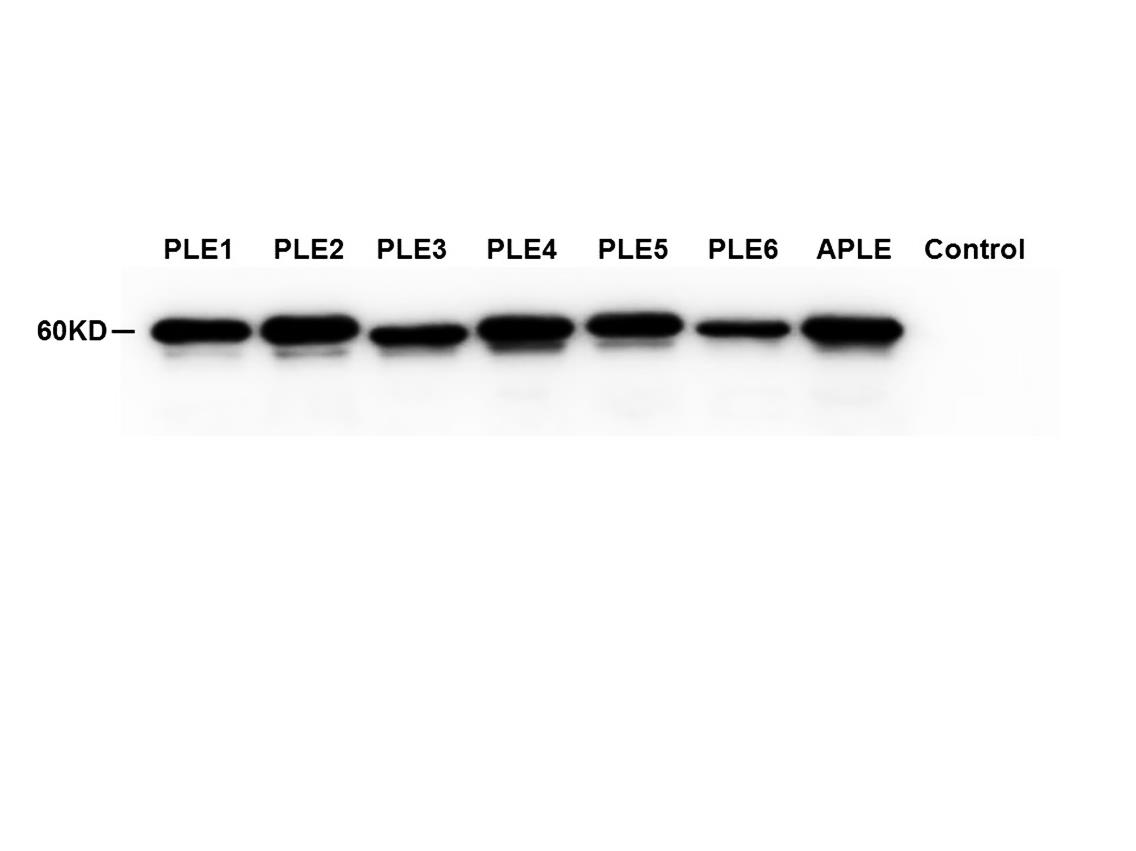
**

**Figure 1. Detection of antibody cross-reactivity against PLEs.**

The following figure was the original image of figure 1.

**
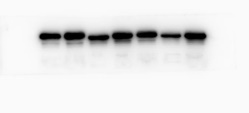
**

Figure S1

**Supplementary Figure S2A**

**
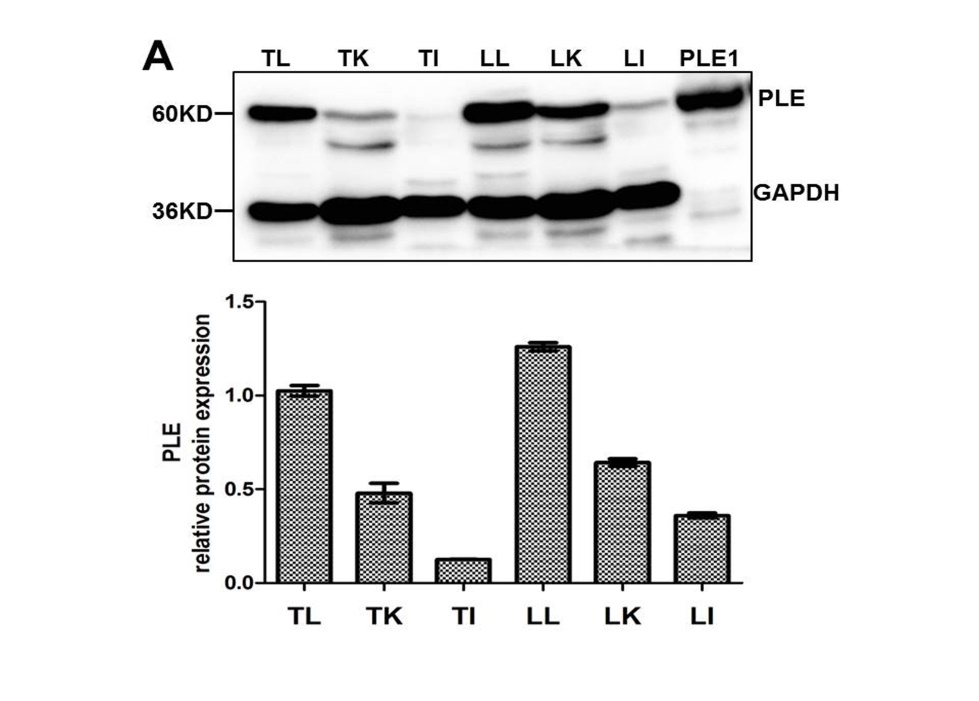
**

**Figure 2. Detection of protein and hydrolytic activities of PLEs and S9 fractions pool**.

In the figure 2A, the grouping of blot was cropped from the red box of the following figure S2A. In the figure S2A, the same recombinant PLE1 (10 μg) was used as the positive control and loaded in last two lanes of the following figure.


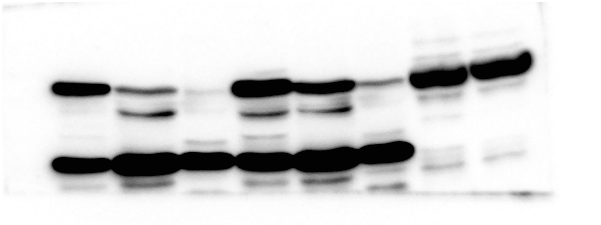


**TL TK TI LL LK LI PLE1 PLE1**

Figure S2A

**Supplementary Figure S3**

**
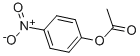
**

Figure S3 The structure of *p*-NPA

**Supplementary Figure S6**


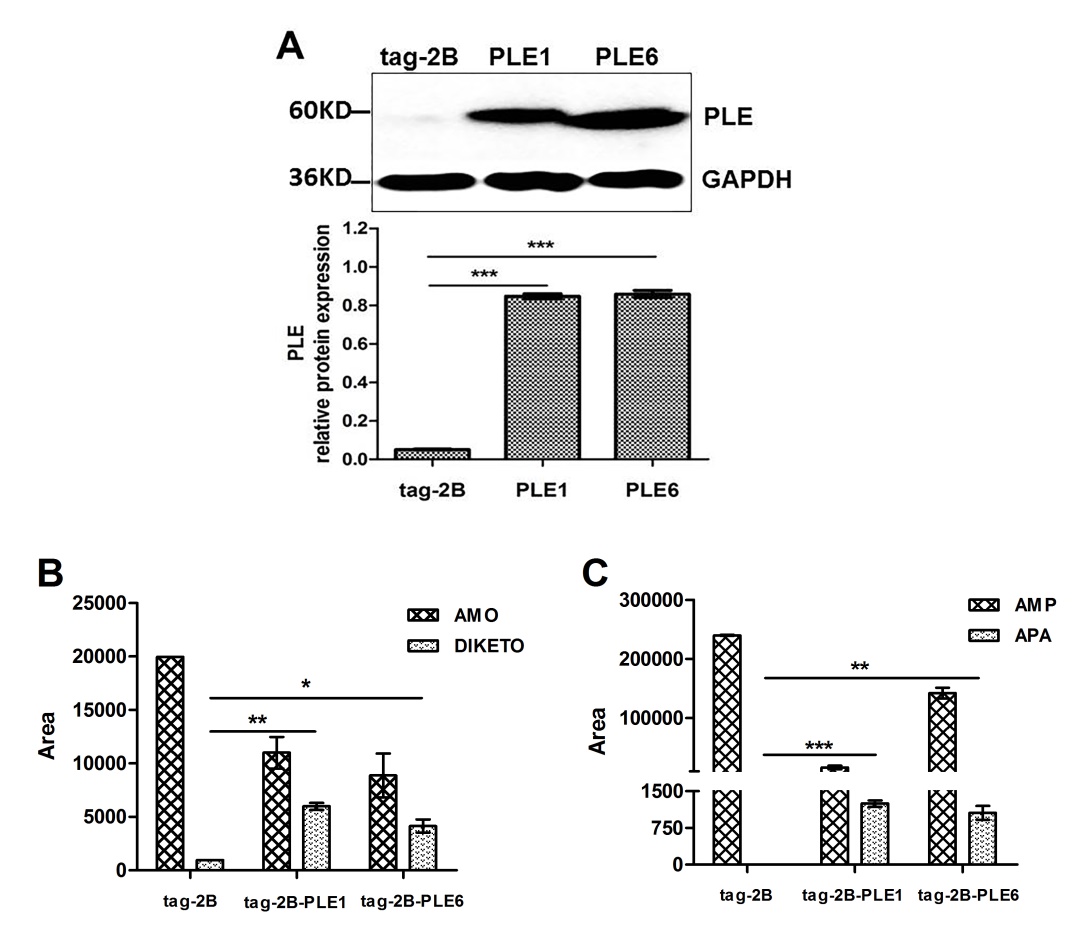


**Figure 6. The hydrolytic activities of PLE1 and PLE6 expressed in 293T cells toward AMO and AMP.**

In the figure 6A, the grouping of blot was cropped from the red box of the following figure S6A.


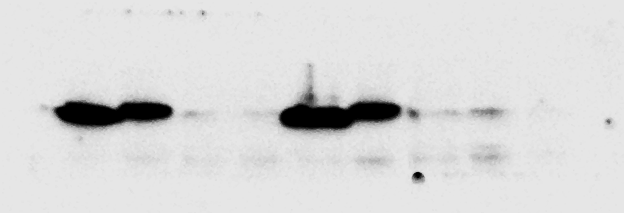


**M Control tag-2B PLE1 PLE6 Control tag-2B PLE1 PLE6**


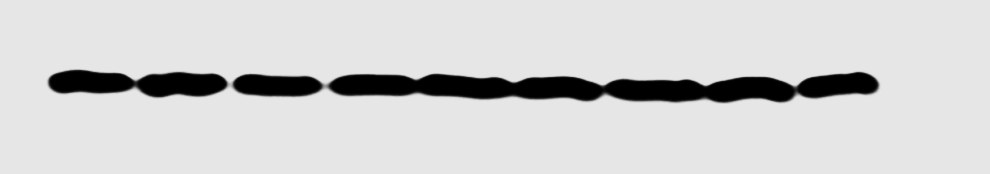


Figure S6A

**Supplementary Figure S7**


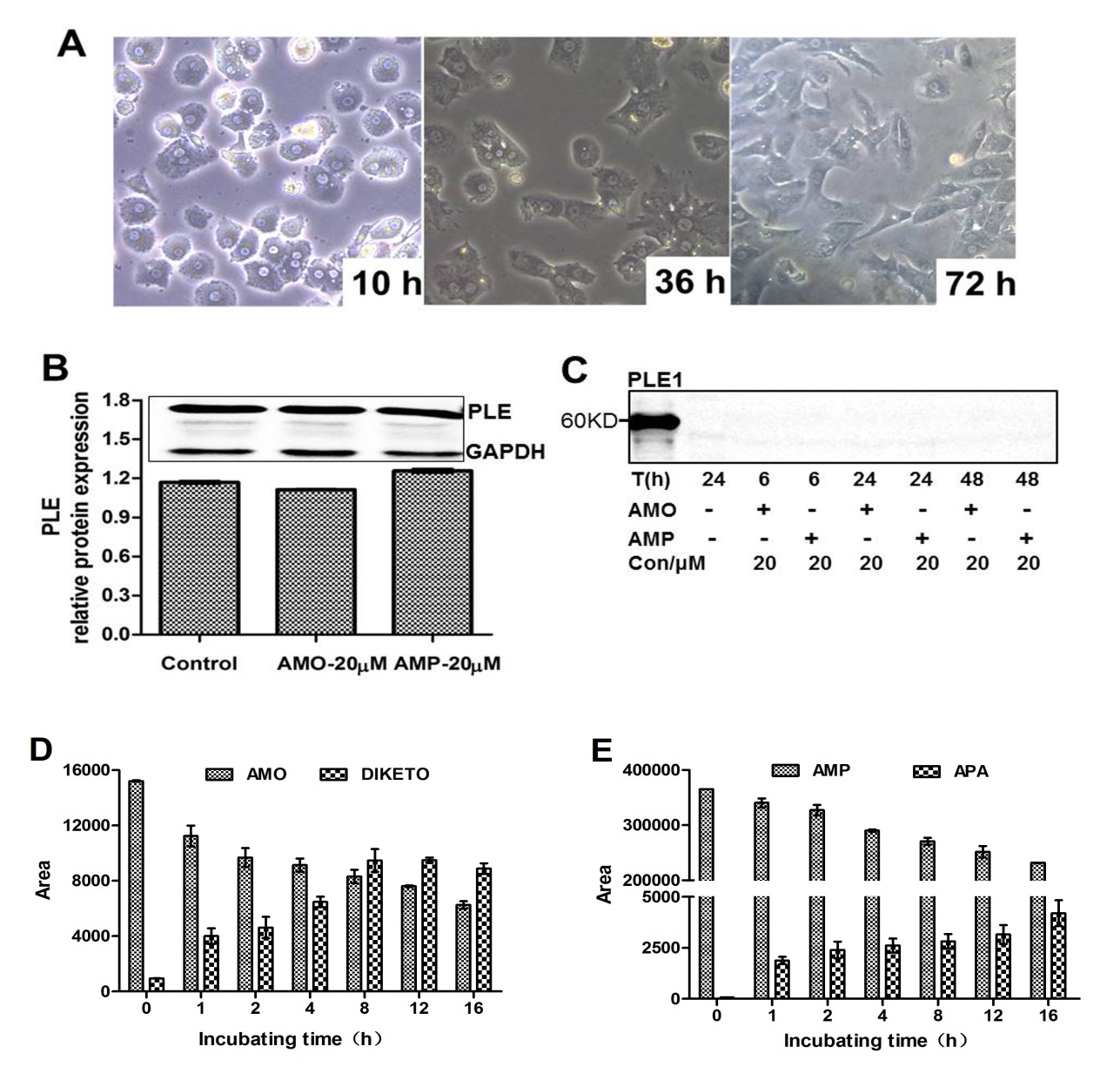


**Figure 7. Detection of PLE isoenzymes and the hydrolytic activities in pig hepatocytes toward AMO and AMP.**

In the figure 7B, the grouping of blot was cropped from the red box of the following figure S7B. In the figure S7B, lane 1 and 2 were the recombinant PLE1 and PLE6 (10 μg) respectively that were used as the positive controls.


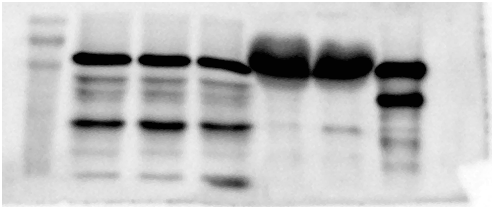


**1 2**

Figure S7B

In the figure 7C, the grouping of blot was cropped from the red box of the following figure S7C. In the figure S7C, lane 1 was the pig hepatocytes lysate and lane 2 was the recombinant PLE1, which were used as the positive controls.


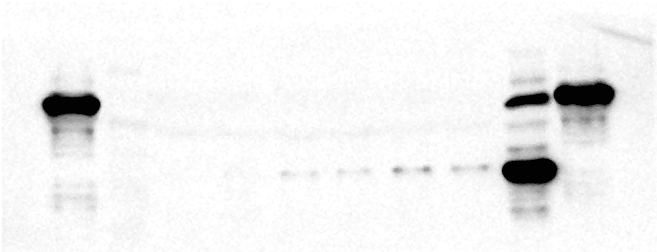


**1 2**

Figure S7C

**Supplementary Figure S8**

**
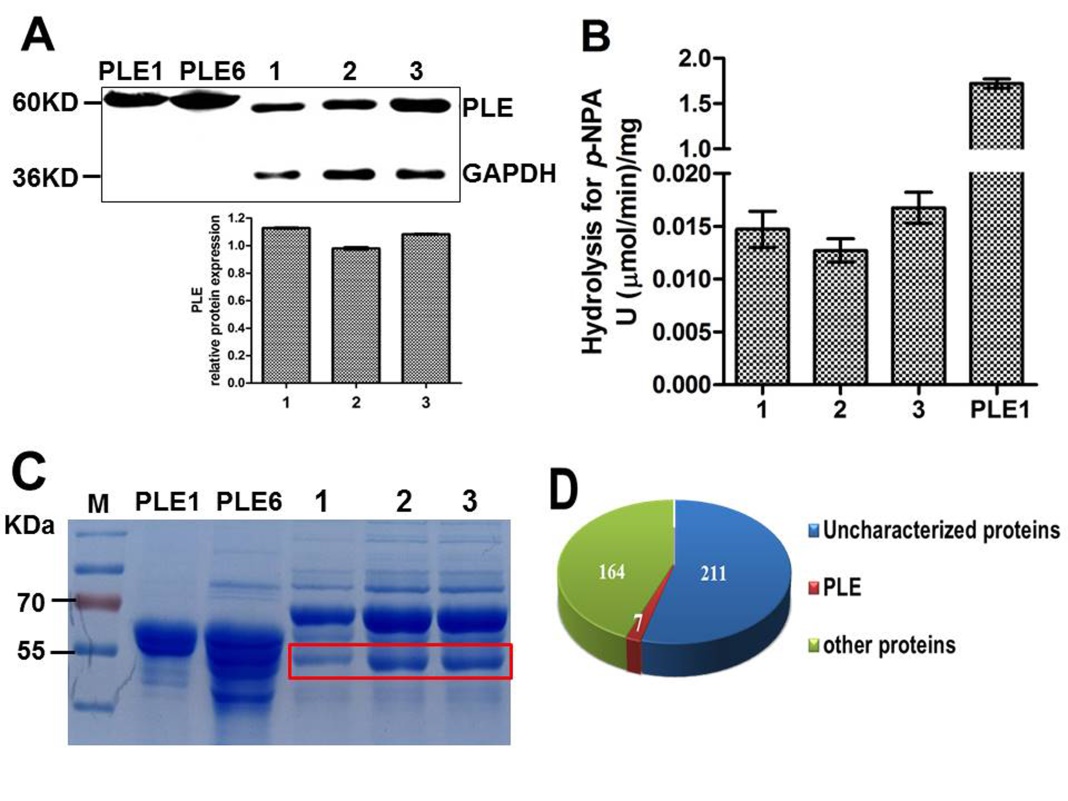
**

**Figure 8. Detection the protein level of PLEs in pig serum and hydrolytic activity for *p*-NPA.**

In the figure 8A, the grouping of blot was cropped from the red box of the following figure S8A. In the figure S8A, the recombinant PLE1 and PLE6 were used as positive controls, lane 1, 2 and 3 were serum pool samples (n=4) from pigs in three different farms respectively and were diluted 100-fold with PBS (50 mM, pH 7.4), lane 4, 5 and 6 were serum pool samples (n=4) from pigs in three different farms respectively and were diluted 25-fold with PBS (50 mM, pH 7.4).


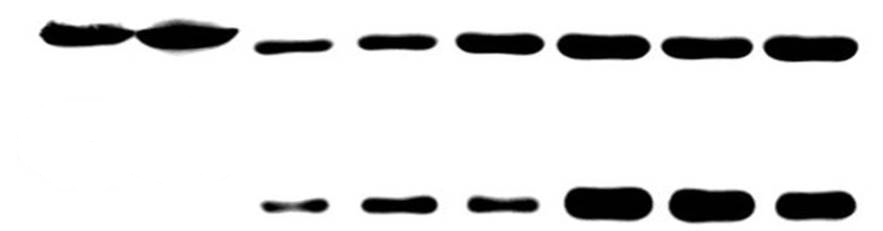


**PLE1 PLE6 1 2 3 4 5 6**

Figure S8A

**Supplementary Table S1**

Table S1 The LC-MS traces and the fragmentation patterns of parent drugs (AMO and AMP)

|  | Amoxicillin (AMO) | Ampicillin (AMP) |
| --- | --- | --- |
|  | C_16_H_19_N_3_O_5_S | C_16_H_19_N_3_O_4_S |
| m/z | 366.112 | 350.118 |
| HPLC | 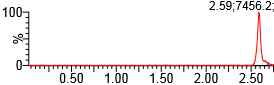 | 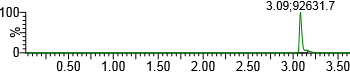 |
| MS | 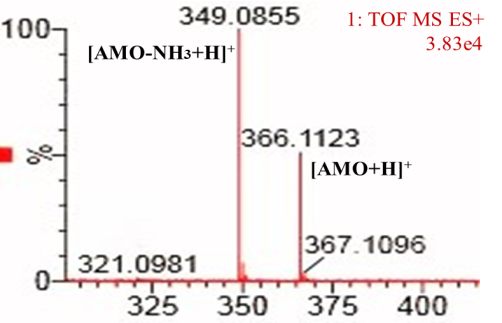 | 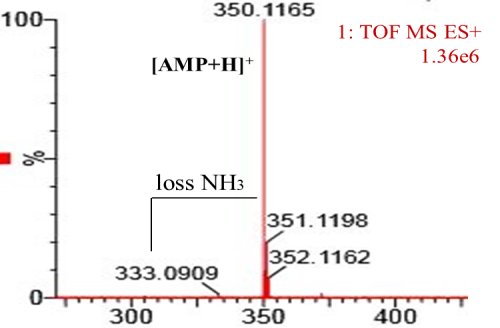 |
| MS/MS | 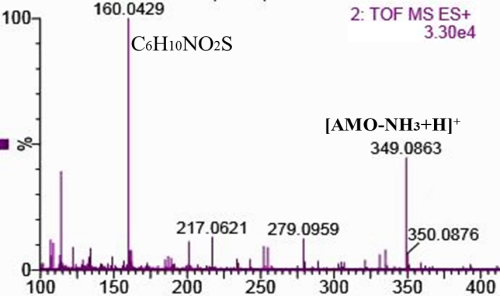 | 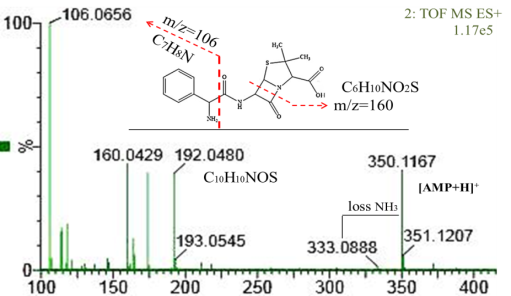 |

Table S2 The LC-MS traces and the fragmentation patterns of hydrolysis products (AMA, DIKETO, APA).

|  | 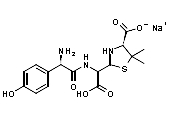AMA (C_16_H_21_N_3_O_6_S) | DIKETO (C_16_H_19_N_3_O_5_S) | 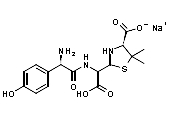APA (C_16_H_21_N_3_O_5_S) |
| --- | --- | --- | --- |
| m/z | 384.1228 | 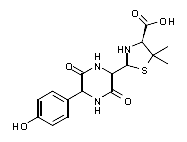366.1125 | 368.1292 |
| Structural formula |  |  |  |
| HPLC | 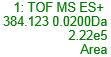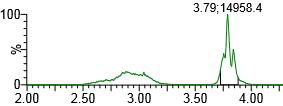 | 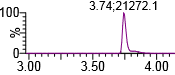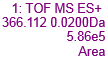 | 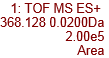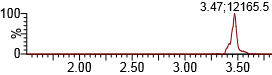 |
| MS | 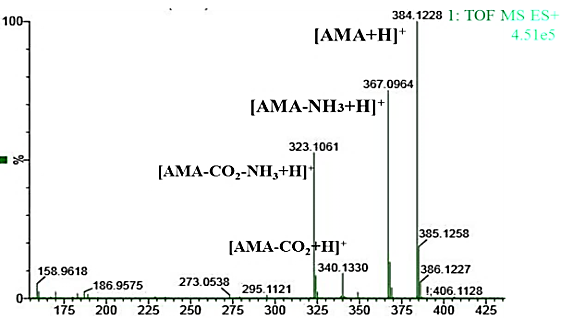 | 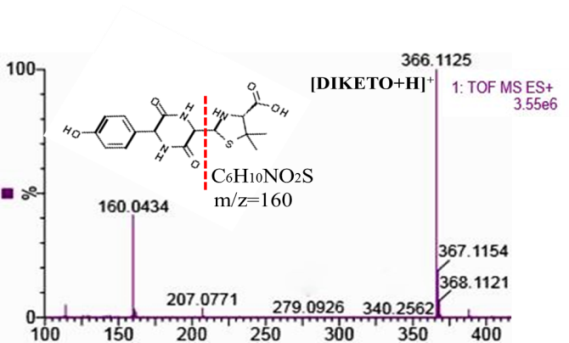 | 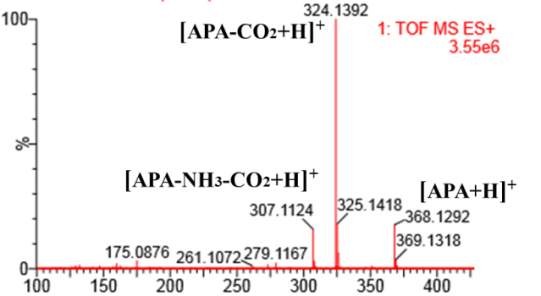 |
| MS/MS | **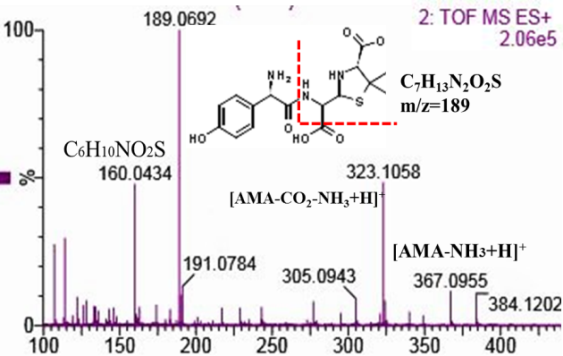** | **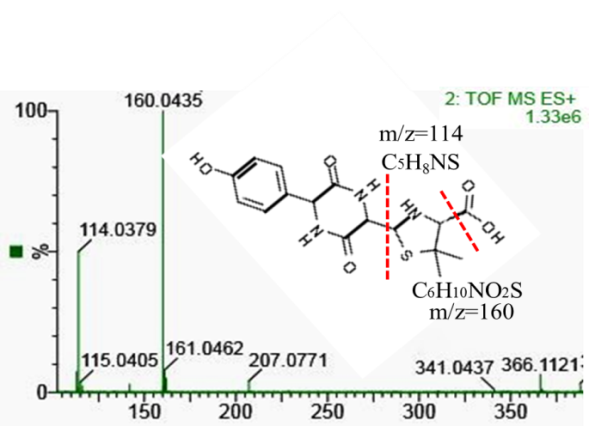** | **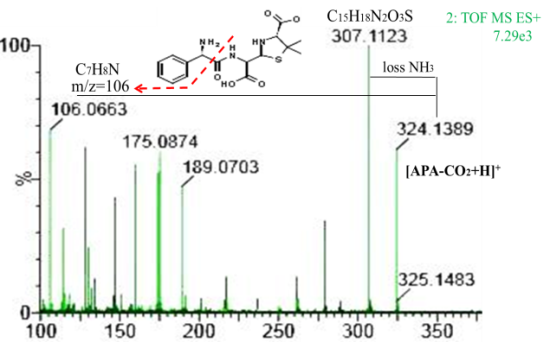** |
